# Factors Associated with Level II and V Involvement in Lateral Neck Dissection for pN1b Papillary Thyroid Carcinoma

**DOI:** 10.3390/cancers18213473

**Published:** 2026-10-28

**Authors:** Patricia Díaz Ríos, Ana Belén Bustos Merlo, Juan Ignacio Arcelus Martínez, Antonio Becerra Massare, Nuria Victoria Muñoz Pérez, Jesús María Villar del Moral

**Affiliations:** 1 General and Digestive Surgery Department, Baza Regional Hospital, Baza, 18800 Granada, Spain; 2 Endocrine Surgery Unit, General and Digestive Surgery Department, Virgen de las Nieves University Hospital, 18014 Granada, Spainnumupe@gmail.com (N.V.M.P.); 3 Department of Surgery, Faculty of Medicine, University of Granada, 18071 Granada, Spain; 4 Instituto de Investigación Biosanitaria ibs. GRANADA, 18012 Granada, Spain

**Keywords:** papillary thyroid carcinoma, lateral neck dissection, lymph node metastases, lymph node ratio, morbidity, risk factors

## Abstract

**Background:** Lateral neck dissection is the standard surgical treatment for patients with papillary thyroid carcinoma (PTC) and lateral cervical lymph node metastases (pN1b). However, the optimal extent of dissection remains debated, particularly for levels II and V. We examined patterns of nodal involvement and factors associated with metastases at these levels. **Methods:** This single-center retrospective study included 70 patients with pN1b PTC who underwent lateral neck dissection between 2019 and 2025. Demographic, clinical, surgical, and pathological variables were analyzed. The lymph node ratio (LNR), defined as metastatic nodes divided by harvested nodes, was dichotomized at the cohort median. Bivariate associations were assessed using Pearson’s χ^2^ test, Fisher’s exact test, or the Fisher–Freeman–Halton exact test, as appropriate; *p* < 0.05 indicated statistical significance. **Results:** Median age was 46 years and 60% were female. Involvement was highest at levels III (82.9%) and IV (65.7%), followed by II (55.7%) and V (23.9%). Median LNR was 0.25. Level II involvement was more frequent in male patients (71.4% vs. 45.2%; *p* = 0.031) and patients with high versus low LNR (76.5% vs. 36.1%; *p* = 0.001). Histological subtype was associated with level V involvement (*p* = 0.027), predominantly in classic PTC. **Conclusions:** Male sex and higher LNR were associated with level II involvement in bivariate analyses; an exploratory association was also observed between histological subtype and level V involvement. These findings do not establish independent predictors or justify altering the extent of neck dissection. Larger cohorts and multivariable analyses should validate these findings.

## Simple Summary

Papillary thyroid carcinoma frequently spreads to cervical lymph nodes. When metastases involve the lateral neck, surgical removal of the affected lymph nodes is generally indicated. However, the optimal extent of lateral neck dissection remains debated, particularly in regions where metastases are less common and surgery may increase the risk of complications. This study evaluated 70 patients with papillary thyroid carcinoma and lateral cervical lymph node metastases to examine the distribution of metastatic involvement and explore factors associated with specific cervical levels. Levels III and IV were the most frequently affected, while level V showed the lowest rate of involvement. Metastases in level II were associated with male sex and a higher overall metastatic lymph node ratio. These findings provide preliminary evidence that may help guide future research toward more individualized surgical approaches.

## 1. Introduction

Papillary thyroid carcinoma (PTC) is the most common type of thyroid cancer. Although it generally has a favorable prognosis, it exhibits a marked propensity for cervical lymphatic dissemination. The presence of lymph node metastases, particularly in the lateral neck compartment, is associated with an increased risk of persistent disease and locoregional recurrence and constitutes a key consideration in risk stratification and treatment planning [1,2,3]. The 2025 American Thyroid Association (ATA) guidelines recommend an individualized approach to the management of differentiated thyroid cancer based on both the characteristics of the primary tumor and the extent of nodal disease [1].

In patients with confirmed lateral cervical lymph node metastases (pN1b), therapeutic lateral neck dissection remains the standard surgical treatment [1]. However, the optimal extent of this procedure remains controversial, particularly with respect to levels II and V. Metastatic involvement varies across cervical levels, with levels III and IV being the most frequently affected, followed by levels II and V [4,5,6]. This distribution pattern was further supported by a recent meta-analysis, which reported a substantially lower prevalence of metastases in sublevels IIb and Va than in IIa and Vb [7]. A separate study specifically examining sublevel IIb further explored its metastatic involvement and relationship to level IIa disease [8].

This heterogeneous metastatic distribution has raised questions about the need to routinely include all lateral neck levels in surgical dissection. Some authors advocate comprehensive dissection of levels II–V to minimize the risk of residual disease and subsequent reoperations [5,9,10,11], whereas others have proposed more selective approaches to reduce surgical morbidity without compromising oncological control [6,12]. Recent studies have also investigated clinicopathological and imaging factors that may help identify patients for whom dissection of certain lateral neck levels, particularly II and V, could potentially be omitted [13,14].

This consideration is especially important for level V, where dissection can increase the risk of shoulder pain and dysfunction due to manipulation of the spinal accessory nerve [12,15]. A meta-analysis comparing selective with more extensive neck dissections reported no significant differences in recurrence rates, although morbidity tended to be higher following more extensive procedures [16]. Retrospective studies comparing the extent of dissection have reported differing recurrence findings, underscoring the need to interpret these comparisons in light of selection and treatment differences [3,10]. Thus, identifying factors associated with metastases at specific cervical levels may contribute to individualizing the extent of lateral neck dissection according to each patient’s risk profile.

Beyond tumor-related clinical and pathological characteristics, the burden of nodal disease may provide further information to guide surgical decision-making. The lymph node ratio (LNR), defined as the ratio of metastatic to total harvested lymph nodes, has been associated with an increased risk of locoregional recurrence in PTC [2,17,18]. Evidence from studies focusing on central compartment dissection also links nodal burden to recurrence, although their LNR definitions and populations differ from those of pN1b lateral neck cohorts [19]. In patients with pN1b disease, Park et al. reported that an LNR > 0.22 was associated with reduced 5-year locoregional recurrence-free survival and remained significantly associated with recurrence in multivariable adjustment [20]. Consistent with these findings, Luo et al., in a cohort of 662 patients with PTC and cervical lymph node metastases, identified a high LNR as an independent factor associated with poorer recurrence-free survival [20]. However, the LNR cutoff values vary considerably across studies [21], and relatively few have investigated whether nodal burden is associated with metastatic involvement at specific cervical levels.

The aim of this study was to characterize the distribution of lateral cervical lymph node metastases in patients with pN1b PTC undergoing lateral neck dissection and to identify clinical and pathological factors associated with metastatic involvement at levels II and V. We further examined the association between metastatic lymph node burden, quantified using the LNR, and metastases at these specific cervical levels. Finally, we characterized the clinicopathological features of the cohort and summarized the available postoperative follow-up findings.

## 2. Materials and Methods

### 2.1. Study Design and Population

This single-center retrospective observational study included 70 patients with histologically confirmed lateral cervical lymph node metastases from papillary thyroid carcinoma (pN1b) who underwent lateral neck dissection between 2019 and 2025.

Data were retrieved from the Eurocrine^®^ database (Eurocrine Society, Vienna, Austria; https://eurocrine.eu/, accessed on 25 October 2026), a prospective, pan-European clinical registry to which our surgical unit has contributed data since 2015.

### 2.2. Inclusion and Exclusion Criteria

Patients with pN1b PTC who underwent lateral neck dissection between 2019 and 2025 were included, whether the procedure was performed concomitantly with initial thyroid surgery or during reoperation for persistent or recurrent disease. Patients who underwent thyroid surgery without lateral neck dissection, with or without concomitant central neck dissection, were excluded. In patients with bilateral lateral cervical lymph node metastases, only the side with the highest absolute number of metastatic lymph nodes was included in the analysis.

### 2.3. Clinical, Surgical and Pathological Variables

Demographic, clinical, surgical, and pathological data were collected. The demographic and clinical variables comprised age, sex, surgical indication, and the timing of lateral neck dissection, distinguishing between procedures performed during initial thyroid surgery and reoperations for persistent or recurrent disease.

Preoperative tumor assessment included the EU-TIRADS ultrasound classification and Bethesda cytological category of the malignant nodule. The pathological characteristics evaluated comprised PTC histological subtype, primary tumor size and location, pathological T stage according to the 8th edition of the TNM classification, multicentricity, extrathyroidal extension, associated lymphocytic thyroiditis, and BRAF mutational status, when available.

The extent of lymph node dissection was documented, including whether concomitant central neck dissection (level VI) was performed, as well as the total numbers of harvested and metastatic lymph nodes. For patients undergoing reoperation, pathological findings from previous central neck dissections were distinguished from those obtained during the procedure being analyzed. This distinction was considered when assessing level VI metastatic involvement and determining the corresponding pathological denominators.

The lymph node ratio (LNR) was calculated as the ratio of metastatic to total harvested lymph nodes, both overall and for each cervical level. For bivariate analyses, the LNR was dichotomized at the cohort-specific median (0.25), yielding two approximately balanced groups (≤0.25 and >0.25) for exploratory comparisons. A sensitivity analysis was also conducted using an alternative LNR cutoff of 0.22, previously reported in the literature, to evaluate the robustness of its association with level II lymph node involvement.

Metastatic involvement was determined by pathological examination and evaluated separately at cervical levels II, III, IV and V. The lateral neck dissection specimen was removed en bloc and subsequently divided intraoperatively by the surgeon into individual cervical levels (II–V) before submission for pathological examination. The surgical procedure was performed using bipolar coagulation, intraoperative neuromonitoring, and magnifying loupes. Cervical level boundaries were defined according to the standardized classification proposed by the American Head and Neck Society (AHNS) [22]. In this study, levels II and V were analyzed in their entirety, without separate assessment of sublevels IIa and IIb or Va and Vb, respectively. The primary study outcomes were the presence or absence of lymph node metastases at levels II and V.

### 2.4. Follow-Up and Oncological Outcomes

Follow-up duration was defined as the interval, expressed in months, between surgery and the last available follow-up visit. Treatments administered during this period were documented, including radioactive iodine therapy, external beam radiotherapy and tyrosine kinase inhibitors (TKIs). At the most recent follow-up visit, response to treatment was classified as excellent, indeterminate, biochemical incomplete or structural incomplete response according to the ATA response-to-therapy criteria [1]. Deaths occurring during the follow-up were also recorded, along with their causes, distinguishing between PTC-related deaths and deaths due to other causes.

### 2.5. Statistical Analysis

Descriptive statistics were used to summarize the study cohort. Categorical variables were reported as absolute frequencies and percentages, while quantitative variables were presented as medians and interquartile ranges (IQRs). Bivariate analyses were conducted to examine associations between clinical and pathological variables and metastatic involvement at levels II and V. Categorical variables were compared using Pearson’s χ^2^ test when the required assumptions were satisfied. Fisher’s exact test was applied to 2 × 2 contingency tables with low expected cell frequencies, whereas the Fisher-Freeman-Halton exact test was employed for contingency tables with more rows or columns.

Multivariable logistic regression was initially considered but was not performed because of the limited number of candidate variables identified in the bivariate analyses and the insufficient number of outcome events to ensure reliable model estimation. A two-sided *p* value < 0.05 was considered statistically significant. All statistical analyses were conducted using IBM SPSS Statistics, version 25.0 (IBM Corp., Armonk, NY, USA).

## 3. Results

### 3.1. Patient Characteristics

Of the 428 patients who underwent surgery for PTC between 2019 and 2025, 70 cases (16.4%) had pN1b disease and underwent lateral neck dissection, thus meeting the study inclusion criteria. The median age was 46 years (IQR, 34–59.7). The cohort comprised 42 women (60%) and 28 men (40%).

**Figure 1:**
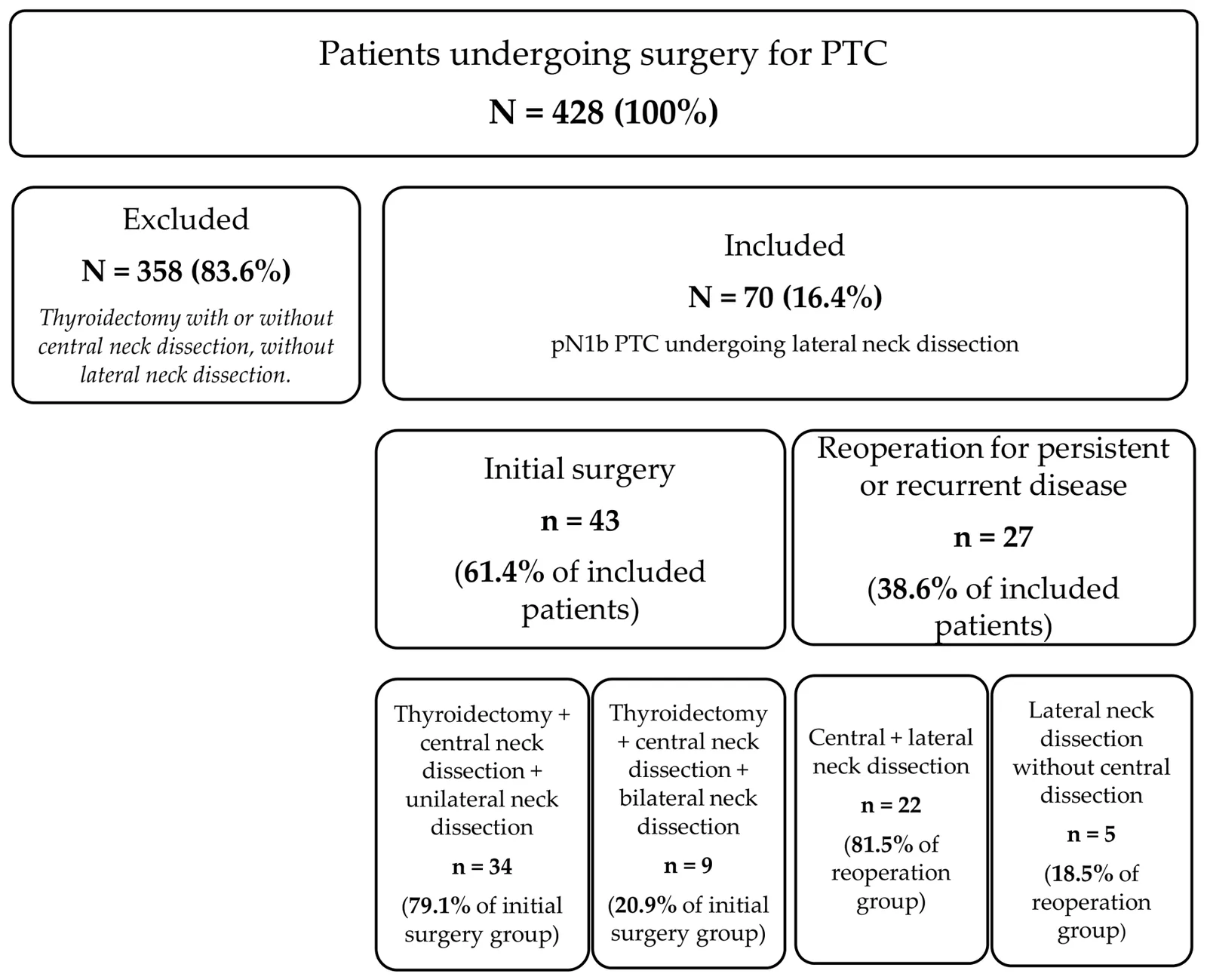
Flow diagram of patient selection and surgical procedure distribution.

In 43 patients (61.4%), lateral neck dissection was performed concomitantly with the initial thyroid surgery, whereas 27 (38.6%) underwent reoperation for persistent or recurrent disease. Figure 1 illustrates the patient selection process and the distribution of surgical procedures according to the surgical setting.

Among patients undergoing reoperation, the median interval between the initial procedure and reoperation was 29 months (IQR, 14–52.5). Completion thyroidectomy was performed concomitantly in seven patients (25.9%). Based on ultrasonographic assessment performed before the initial thyroid surgery, thyroid nodules were classified as EU-TIRADS 4 in 17 cases (24.3%), and as EU-TIRADS 5 in 38 cases (54.3%), whereas EU-TIRADS classification was unavailable in 15 cases (21.4%).

Regarding preoperative cytology, 6 patients (8.6%) had Bethesda category II in nodules subsequently confirmed to be malignant on histopathological examination, 17 (24.3%) had Bethesda category V, and 43 (61.4%) had Bethesda category VI. Cytological data were unavailable in four cases (5.7%) (Table 1).

### 3.2. Pathological Characteristics

The most common histological subtype was classic PTC, identified in 48 patients (68.6%), followed by the follicular variant in 11 (15.7%). Aggressive histological variants, comprising the diffuse sclerosing and tall-cell variants, were identified in ten patients (14.3%). In one patient (1.4%), no primary tumor was identified in the thyroid gland.

Multicentricity was identified in 30 of the 69 patients with an intrathyroidal tumor (43.5%). Tumor size data were available for 65 patients, with a median tumor diameter of 20 mm (IQR, 14–32). According to the 8th edition of the TNM classification, pathological T stages were distributed as follows: pT0 in one patient (1.4%), pT1a in 12 (17.1%), pT1b in 17 (24.3%), pT2 in 13 (18.6%), pT3a in 6 (8.6%), pT3b in 14 (20.0%), pT4a in 5 (7.1%), and pT4b in one (1.4%). Pathological T stage could not be determined in one patient (pTX, 1.4%).

Regarding the anatomical location of the largest primary tumor, 16 patients (22.9%) had tumors located in the upper pole, nine (12.9%) in the middle third, nine (12.9%) in the lower pole, seven (10.0%) with involvement of an entire thyroid lobe, three (4.3%) in the isthmus, and one (1.4%) with extensive thyroid involvement. Tumor location was not documented in 25 patients (35.7%) (Table 2).

Extrathyroidal extension was observed in 21 of the 68 patients with available information (30.9%), and concomitant lymphocytic thyroiditis was present in 14 patients (20%). BRAF mutational status was available for 25 patients, of whom 16 (64%) harbored a BRAF mutation (Table 2).

### 3.3. Pattern of Lymph Node Involvement

The median number of harvested lymph nodes per patient was 46 (IQR, 36–59.5), while the median number of metastatic lymph nodes was 10.5 (IQR, 5.25–17.7). The median overall LNR was 0.25 (IQR, 0.1–0.4).

**Figure 2:**
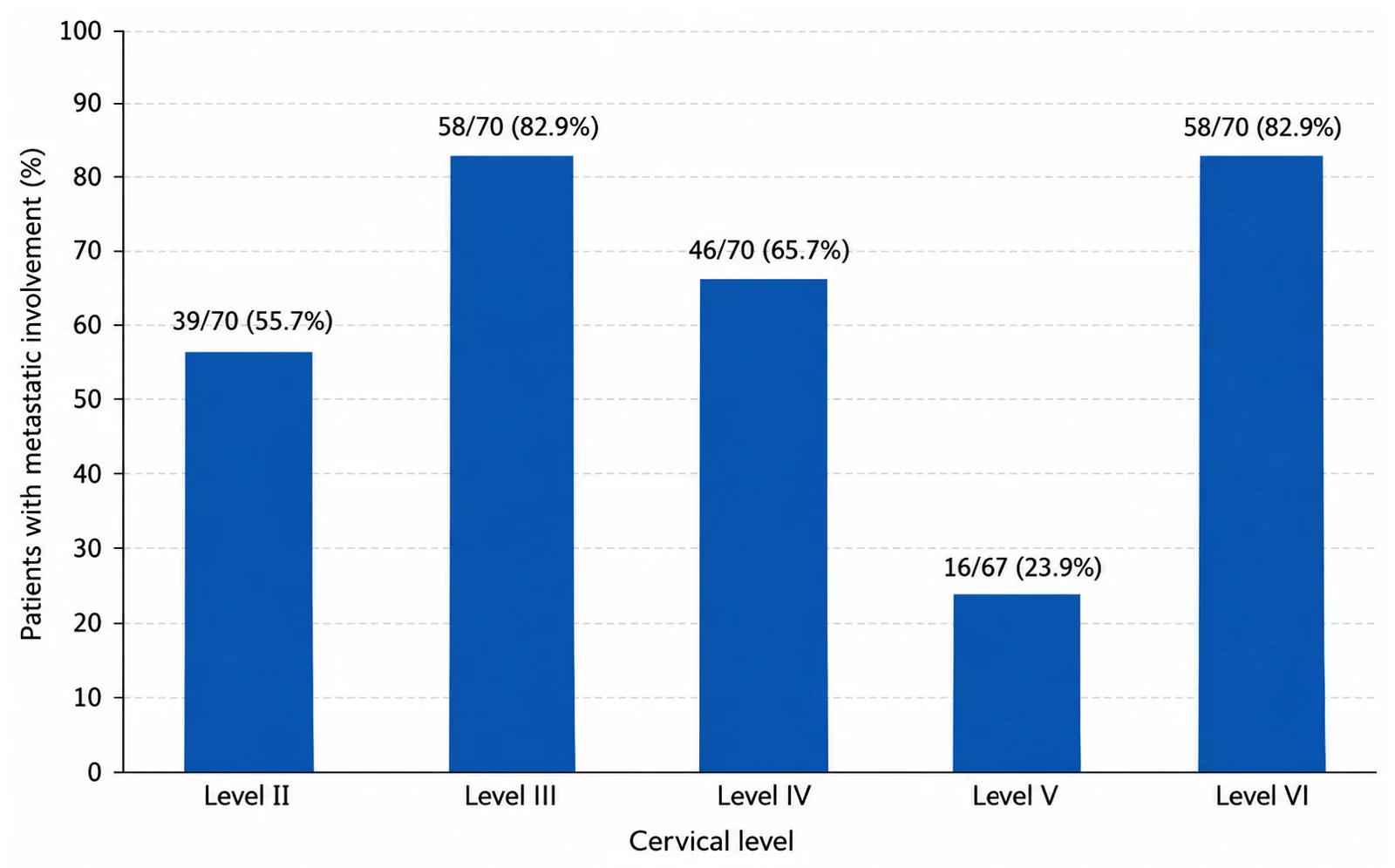
Distribution of metastatic involvement according to cervical level.

The distribution of metastases was heterogeneous across cervical lymph node levels, with levels III and IV being the most frequently involved levels. Level II metastases were identified in 39 patients (55.7%), level III in 58 (82.9%), level IV in 46 (65.7%) and level V in 16 of 67 patients (23.9%). Level V was not dissected in three patients; consequently, pathological findings for this level were available in 67 of the 70 patients included in the study. The central compartment (level VI) showed pathologically confirmed metastatic involvement in 58 patients of the 70 patients (82.9%) based on findings from both the index procedure and previous surgical procedures, where applicable (Figure 2).

Regarding the central compartment (level VI), concomitant central neck dissection was performed in 65 of the 70 patients (92.9%), whereas the remaining five patients (7.1%) underwent lateral neck dissection alone. Among those undergoing concomitant central neck dissection, 54 (83.1%) had metastatic involvement of level VI, whereas 11 (16.9%) had no metastases identified in this compartment. Of the five patients who underwent lateral neck dissection alone, four had level VI metastases documented during a previous surgical procedure, while no level VI metastases had been documented in the remaining (Table 3).

### 3.4. Factors Associated with Level II and V Involvement

As shown in Table 4, metastatic involvement of level II was significantly more frequent in male patients than in female patients (71.4% vs. 45.2%, *p* = 0.031). Level II involvement was also significantly associated with a higher global LNR, after dichotomization at the cohort median of 0.25. No statistically significant associations were observed between level II involvement and the remaining clinical or pathological variables analyzed.

To assess the robustness of this association, a sensitivity analysis was performed using an alternative LNR cutoff of 0.22, as previously reported by Park et al. [20]. The association between a higher LNR and level II involvement remained statistically significant, with metastases identified in 28 of 38 patients (73.7%) with an LNR > 0.22, compared with 11 of 32 patients (34.4%) with an LNR ≤ 0.22 (OR = 5.35, 95% CI: 1.92–14.92; *p* = 0.0016, Fisher’s exact test).

Metastatic involvement of level V was observed in 16 of the 67 evaluable patients (23.9%). In bivariate analysis, histological subtype was significantly associated with level V involvement (*p* = 0.027). Level V metastases were identified in 15 of 45 patients (33.3%) with classic PTC, compared with none of the 11 patients with the follicular variant and one of the 10 patients (10%) with aggressive variants. Histological subtype information was unavailable for one patient; therefore, the analysis of the association between histological subtype and level V involvement was based on 66 patients with complete data for both variables. No statistically significant associations were identified for the remaining clinical or pathological variables analyzed (Table 5).

### 3.5. Follow-Up

The median follow-up duration from lateral neck dissection to the last available follow-up visit was 56.5 months (IQR, 17.5–86.5). During follow-up, 65 patients (92.9%) received radioactive iodine therapy, 2 (2.9%) received external beam radiotherapy and 3 (4.3%) received treatment with TKIs.

At the last available follow-up, according to the response-to-therapy classification [1], 49 patients (70%) achieved an excellent response, 11 (15.7%) had an indeterminate response, 5 (7.1%) had a biochemical incomplete response and 5 (7.1%) had a structural incomplete response. All five patients with a structural incomplete response had distant metastatic disease, with no evidence of persistent or recurrent locoregional disease. Of these, three patients (60%) had pulmonary metastases only, whereas two patients (40%) had both pulmonary and bone metastases.

During the follow-up period, two patients (2.9%) died, both from causes unrelated to PTC.

## 4. Discussion

In our cohort, levels III and IV were the most frequently involved, with metastatic involvement rates of 82.9% and 65.7%, respectively, followed by level II (55.7%), whereas level V metastases were present in 23.9% of evaluable patients. Previous studies have similarly reported a predilection of lateral cervical PTC metastases for levels III and IV [4,5,6,23]. In the meta-analysis by Eskander et al., the rates of involvement of levels II, III, IV, and V were 53.4%, 70.5%, 66.3%, and 25.3%, respectively, closely resembling those observed in our cohort [4]. Kang et al. also reported comparable rates of level II (53.6%) and V (25.4%) involvement among 209 patients with metastatic PTC [13]. Hartl et al. described a similar distribution with frequent involvement of levels III and IV and a lower prevalence of level V metastases, particularly in sublevel Va [7]. More recently, Ozel et al. reported level II involvement in 58.9% of comprehensive lateral neck dissections [14]. Taken together, these studies suggest a relatively consistent pattern of lateral cervical lymph node involvement in PTC across different cohorts.

One of the main findings of our study was the association between level II involvement and a higher metastatic lymph node burden, as reflected by the LNR. Patients with an LNR above the cohort median (0.25) had a significantly higher frequency of level II metastases than those with an LNR equal to or below this threshold. The LNR has gained increasing interest as a marker of nodal disease burden and as a potential prognostic factor in PTC [18]. Park et al., in patients with N1b disease, reported that an LNR > 0.22 was associated with lower 5-year locoregional recurrence-free survival [17]. Similarly, Luo et al. identified a high lateral neck LNR as an independent factor associated with poorer recurrence-free survival [20].

Regarding the anatomical distribution of nodal metastases, Ozel et al. found that both a lateral neck LNR > 0.10 and more than five metastatic lateral lymph nodes were significantly associated with level II involvement in univariate analysis [14]. Despite differences in LNR thresholds and definitions across studies, these findings are broadly consistent with the association observed in our cohort between higher nodal burden and level II involvement.

Although previous studies primarily investigated the prognostic value of the LNR, these findings, together with our results, suggest that the LNR may also reflect the anatomical extent of nodal disease. Reported LNR cutoffs vary considerably across prognostic studies [18,21]. In our study, the median LNR was used to divide the cohort into two approximately balanced groups for exploratory comparisons, yielding a cutoff value of 0.25. Although this value was also previously reported in the literature, in our study, it was derived from the distribution of LNR within the cohort rather than from ROC curve analysis or external validation. Therefore, it should not be interpreted as a validated threshold for high-risk disease. Furthermore, the association between higher LNR and level II involvement remained statistically significant when an alternative cutoff of 0.22 was applied, as proposed by Park et al. [17], supporting the robustness of this finding across the two thresholds examined.

Nevertheless, neither cutoff can be assumed to be generalizable to other patient populations without external validation. In addition, because LNR is a postoperative pathological parameter requiring knowledge of both harvested and metastatic lymph nodes, it cannot directly inform preoperative decisions regarding the extent of neck dissection. Accordingly, the observed association should be considered exploratory rather than a basis for surgical decision-making.

Level II involvement was also significantly more frequent in male than in female patients. Male sex has previously been associated with higher-risk features and poorer oncological outcomes in some PTC series, and Park et al. also identified sex as a variable associated with locoregional recurrence in pN1b PTC [17]. However, the specific association between male sex and level II involvement has been less extensively studied. Previous studies have also reported associations between level II metastases and larger primary tumor size, upper-pole location, vascular invasion, central lymph node metastatic burden and involvement of levels III and IV [23,24].

The frequency of level II involvement in our cohort (55.7%) was higher than that reported by Huang et al. (34.1%) [25]. This difference may reflect variations in patient selection, study populations and the anatomical classification of cervical lymph node levels. In our study, lymph node specimens were assigned to individual cervical levels intraoperatively by the surgeon, which may have introduced variability in the classification of nodes located near anatomical boundaries. Nevertheless, the contribution of these methodological differences to the observed discrepancy remains uncertain.

Ozel et al. identified extrathyroidal extension and a largest metastatic lateral lymph node size > 2 cm as independent predictors of both level II and Vb involvement [14]. In contrast, extrathyroidal extension was not associated with level II involvement in our cohort. Differences in sample size, patient characteristics and the variables assessed may account for some of the discrepancies between studies. Overall, the association between male sex and level II involvement observed in our cohort warrants cautious interpretation and confirmation in larger and independent cohorts.

Level V involvement was considerably less frequent, occurring in almost a quarter of our evaluable patients. This finding is consistent with previous studies reporting a prevalence of level V metastases of approximately 20–25% [4,7,16]. Other cohorts have reported different frequencies, including 28.8% in a series of 330 patients and approximately 12% among patients with solitary pTC, emphasizing the importance of differences in case selection [26,27]. The routine inclusion of level V in lateral neck dissection remains controversial [11]. While some authors advocate dissection of levels II and V to minimize the risk of residual disease [5,9], others have proposed more selective approaches because of the lower frequency of level V involvement and the potential morbidity associated with its dissection [6,12]. Kim et al. reported a higher incidence of shoulder dysfunction related to manipulation of the spinal accessory nerve when level V was included in the dissection [12], while the meta-analysis by Won et al. found no significant differences in recurrence between selective and more extensive neck dissections, although shoulder morbidity tended to be greater with the latter [16].

Regarding factors associated with level V metastases, Kang et al., in a study of 209 patients assessing cervical lymph node involvement at the whole-level scale, identified macroscopic extranodal extension (ENE) as a predictor of level V metastasis in multivariable analysis [13]. In our cohort, extrathyroidal extension (ETE) of the primary tumor was not significantly associated with level V involvement. However, ENE and ETE represent distinct pathological variables: ENE refers to tumor extension beyond the capsule of a metastatic lymph node, whereas ETE describes extension of the primary tumor beyond the thyroid gland. As ENE was not systematically documented in our pathological records, we were unable to assess whether the association reported by Kang et al. was also present in our cohort.

Zhang et al. identified ETE, whole-thyroid tumor location, and simultaneous multilevel involvement as factors associated with level V metastases, whereas Yang et al. found contralateral central nodal metastases to be independently associated with level V involvement in solitary PTC [26,27]. In a cohort of 252 patients, Li et al. reported associations of level V metastases with primary tumor size, central metastatic node count, level II metastases, and BRAF V600E status in multivariable analysis. Notably, the reported association with BRAF mutation was inverse (OR 0.439), highlighting the need for cautious interpretation and external validation [28]. More recently, Ozel et al. reported a low prevalence of level Vb involvement and found that most patients with Vb metastases had extensive multilevel lateral neck disease. Based on these findings, they suggested that level Vb dissection might be omitted in selected patients with lateral lymph nodes smaller than 2 cm, absence of multilevel involvement, and negative preoperative ultrasonography [14].

In our cohort, an association between histological subtype and level V involvement was observed in the bivariate analysis. Among the 66 patients with available histological subtype information, level V involvement was observed in 15 of 45 patients with classic PTC, none of the 11 patients with follicular variant PTC and only one of the 10 patients with aggressive histological variants. However, the small number of level V metastatic events (16 of 67 evaluable patients) and their uneven distribution across histological subtypes limit the reliability of this association, which may reflect random variation rather than a reproducible biological relationship. Furthermore, our analysis considered level V, without distinguishing between sublevels Va and Vb, limiting direct comparison with studies specifically evaluating level Vb.

Previous studies have identified other factors associated with level V metastasis, particularly ETE or ENE, level IV involvement and simultaneous involvement of multiple lateral neck levels [29]. Associations with nodal burden, including the number of metastatic lymph nodes and the level II LNR, have also been reported [30]. Taken together, these considerations indicate that our finding should be regarded as exploratory and hypothesis-generating, without sufficient evidence to support clinical decision-making or the omission of level V based on histological subtype. Larger, preferably multicenter studies are needed to validate this association and assess the contribution of histological subtype alongside other clinical, imaging, pathological and nodal characteristics.

Our study has several limitations. First, its retrospective, single-center design may introduce selection bias and limit the generalizability of the findings. Second, the sample size, particularly the small number of patients with level V involvement, limits the statistical power to detect associations with level V metastases. Furthermore, level V was not dissected in three patients, reducing the number of evaluable patients to 67. As the decision to omit level V dissection may have been influenced by preoperative or intraoperative findings, selection bias cannot be excluded.

The inclusion of patients undergoing lateral neck dissection during initial surgery alongside those undergoing reoperation for persistent or recurrent disease also introduces heterogeneity into the cohort. Although surgical setting was not significantly associated with level II or V involvement in the bivariate analyses, the limited sample size reduces the statistical power to detect differences between these subgroups. Moreover, differences in previous surgical treatment and disease characteristics may introduce confounding, and the absence of statistically significant associations should not be interpreted as evidence of equivalence between surgical settings.

Finally, BRAF mutational status was available for only 25 of the 70 patients, substantially limiting the statistical power to detect potential associations with level-specific metastatic involvement. Consequently, the lack of statistically significant associations involving BRAF should not be interpreted as evidence that no such associations exist. Although molecular associations with cervical nodal metastases have been investigated in meta-analyses [31], evidence specific to level V remains heterogeneous [28] and our incomplete molecular data do not permit a reliable level-specific assessment.

Another potential limitation is the intraoperative division of the en bloc lateral neck dissection specimen into individual cervical levels by the surgeon. Although the anatomical boundaries were defined according to a standardized classification, this procedure may introduce subjectivity when assigning lymph nodes located near the boundaries between adjacent levels, particularly between levels II and III or IV and V. Such misclassification could affect the accuracy of level-specific metastatic involvement estimates and potentially obscure true associations, complicating the interpretation of non-significant findings. However, given the retrospective nature of the study and the absence of an independent assessment of intraoperative specimen regionalization, the extent of this potential bias could not be reliably quantified.

Furthermore, levels II and V were analyzed as whole levels without distinguishing between sublevels IIa/IIb and Va/Vb, preventing assessment of the distribution of metastases within these sublevels. Consequently, our findings cannot inform the ongoing debate regarding the potential omission of sublevels IIb or Vb during lateral neck dissection. Finally, macroscopic ENE of metastatic lymph nodes was not systematically documented in the pathological reports and therefore could not be analyzed as a potential factor associated with level-specific metastatic involvement.

Primary tumor location was incompletely documented, and some tumors involved multiple anatomical regions. In patients with multifocal disease, the recorded location of the largest tumor may not adequately represent the anatomical distribution of all tumor foci, limiting reliable comparisons based on tumor location within the thyroid gland. Consequently, the substantial proportion of missing data and the heterogeneity of the recorded anatomical categories precluded reliable bivariate analyses according to individual thyroid poles. The maximum diameter of metastatic lymph nodes was not consistently documented in the pathological reports throughout the study period, precluding a reliable determination of the proportion of patients with metastatic lymph nodes larger than 3 cm.

Finally, all identified associations should be considered exploratory and hypothesis-generating, as they were derived from bivariate analyses and cannot establish independent predictive factors. In particular, the associations between male sex or higher LNR and level II involvement may be influenced by confounding, including a potential relationship between sex and overall nodal disease burden, which could not be assessed through multivariable adjustment. Moreover, level II metastases were also observed in a substantial proportion of female patients and those with a lower LNR, precluding the identification of a subgroup in which level II dissection could be safely omitted.

Despite these limitations, our findings provide additional information on the distribution of lateral cervical lymph node metastases in pN1b PTC and highlight the need for further investigation into the potential role of individualized surgical approaches. The association between a higher LNR and level II involvement suggests that metastatic lymph node burden may reflect the anatomical extent of nodal disease, while the observed association between histological subtype and level V involvement warrants further investigation. Nevertheless, these exploratory findings are insufficient to justify changes in the extent of lateral neck dissection. Future multicenter studies with larger cohorts and appropriately powered multivariable analyses are needed to determine whether clinical, imaging, pathological, molecular and genetic tumor characteristics can reliably identify patients who may benefit from more individualized lateral neck dissection strategies.

## 5. Conclusions

In patients with pN1b PTC, lateral cervical lymph node involvement showed a heterogeneous distribution, with levels III and IV being the most frequently involved and level V the least affected. In bivariate analyses, male sex and higher LNR were associated with level II involvement, while an association was observed between histological subtype and level V involvement. However, these findings should be considered exploratory and hypothesis-generating because of the limited sample size, the small number of level V metastatic events and the absence of multivariable adjustment. Furthermore, the cohort-specific LNR cutoff requires external validation before it can be applied to other patient populations. Consequently, these associations cannot be considered independent predictive factors or used to justify changes in the extent of lateral neck dissection. Further research is needed to determine whether more selective surgical approaches can be safely implemented in specific patient subgroups, ideally through larger, multicenter studies incorporating appropriate multivariable analyses and external validation.

## Figures and Tables

**Table 1:** Demographic and clinical characteristics of the study cohort.

Demographic and Clinical Characteristics	Value
Patients, *N*	70
Age, years, median (IQR)	46 (34–59.7)
Sex	
Female	42 (60%)
Male	28 (40%)
Timing of lateral neck dissection	
Initial surgery	43 (61.4%)
Reoperation for persistent/recurrent disease	27 (38.6%)
Time to reoperation, months, median (IQR) *	29 (14–52.5)
Preoperative cytology (Bethesda category)	
Bethesda II	6 (8.6%)
Bethesda V	17 (24.3%)
Bethesda VI	43 (61.4%)
Not available	4 (5.7%)
Preoperative ultrasound (EU-TIRADS)	
EU-TIRADS 4	17 (24.3%)
EU-TIRADS 5	38 (54.3%)
Not available	15 (21.4%)

* Calculated among patients undergoing reoperation (*n* = 27). Values are presented as *n* (%) unless otherwise indicated. IQR, interquartile range; EU-TIRADS, European Thyroid Imaging Reporting and Data System.

**Table 2:** Histopathological characteristics of the study cohort.

Histopathological Characteristics	Value
Histological subtype, *n* (%)	
Classic PTC	48 (68.6%)
Follicular variant	11 (15.7%)
Aggressive variants *	10 (14.3%)
No intrathyroidal tumor identified	1 (1.4%)
Tumor size, mm, median (IQR) †	20 (14–32)
Pathological T stage (TNM 8th edition), *n* (%)	
pT0	1 (1.4%)
pT1a	12 (17.1%)
pT1b	17 (24.3%)
pT2	13 (18.6%)
pT3a	6 (8.6%)
pT3b	14 (20%)
pT4a	5 (7.1%)
pT4b	1 (1.4%)
pTx	1 (1.4%)
Primary tumor location, *n* (%)	
Upper pole	16 (22.9%)
Middle third	9 (12.9%)
Lower pole	9 (12.9%)
Entire thyroid lobe	7 (10%)
Isthmus	3 (4.3%)
Extensive thyroid involvement	1 (1.4%)
Not recorded	25 (35.7%)
Multicentricity *n*/*N* (%) ‡	30/69 (43.5%)
Extrathyroidal extension, *n*/*N* (%) ‡	21/68 (30.9%)
Lymphocytic thyroiditis, *n* (%)	14 (20%)
BRAF mutation, *n*/*N* (%) §	16/25 (64%)

* Diffuse sclerosing and tall-cell variants. † Tumor size was available for 65 patients. ‡ Based on available cases only. The corresponding denominators are provided for each variable. § BRAF status was analyzed based on available cases only (*n* = 25). Note: Primary tumor location refers to the largest tumor focus. In patients with multifocal or extensive disease, this variable may not fully represent the anatomical distribution of the primary tumor. For variables with missing data, percentages were calculated using the number of patients with available information.

**Table 3:** Central neck dissection and level VI metastatic involvement in the study cohort.

Central Neck Dissection and Level VI Status	Value
Central neck dissection during the index procedure (*n* = 70)	
Concomitant central and lateral neck dissection	65 (92.9%)
Only lateral neck dissection	5 (7.1%)
Level VI metastatic involvement across surgical history (*n* = 70)	
Positive	58 (82.9%)
No metastases documented	12 (17.1%)
Level VI metastatic involvement in concomitant central neck dissection (*n* = 65)	
Positive	54 (83.1%)
No metastases documented	11 (16.9%)

Note: Level VI metastatic involvement across surgical history was determined from pathological findings obtained during the index procedure or previous surgical procedures, where applicable. In contrast, level VI involvement in concomitant central neck dissection refers exclusively to specimens obtained during the index procedure. Percentages were calculated using the denominators indicated for each group.

**Table 4:** Factors associated with metastatic involvement of level II.

Variable	Category	Level II Involvement, *n*/*N* (%)	*p*-Value
Sex	Female	19/42 (45.2%)	**0.031**
	Male	20/28 (71.4%)	
Age	≤45 years	21/35 (60%)	0.470
	>45 years	18/35 (51.4%)	
Surgical setting	Initial surgery	25/43 (58.1%)	0.606
	Reoperation	14/27 (51.9%)	
Bethesda category †	II	5/6 (83.3%)	0.321
	V	8/17 (47.1%)	
	VI	23/43 (53.5%)	
EU-TIRADS †	4	9/17 (52.9%)	0.983
	5	20/38 (52.6%)	
Histological subtype †	Classic PTC	28/48 (58.3%)	0.317
	Follicular variant	4/11 (36.4%)	
	Aggressive variants	7/10 (70%)	
BRAF status §	Mutated	9/16 (56.3%)	1.000
	Wild-type	5/9 (55.6%)	
Multicentricity †	No	21/39 (53.8%)	0.609
	Yes	18/30 (60%)	
Extrathyroidal extension †	No	27/47 (57.4%)	0.981
	Yes	12/21 (57.1%)	
Lymphocytic thyroiditis	No	33/56 (58.9%)	0.279
	Yes	6/14 (42.9%)	
Global LNR	≤0.25	13/36 (36.1%)	**0.001**
	>0.25	26/34 (76.5%)	

† Analyses were performed using available data for each variable; therefore, denominators may differ. § BRAF status was analyzed based on available cases only (*n* = 25). Note: Values are *n*/*N* (%) unless otherwise indicated. *p*-values were calculated using Pearson’s χ^2^ test, Fisher’s exact test or Fisher-Freeman-Halton exact test, as appropriate. Bold values indicate statistical significance (*p* < 0.05).

**Table 5:** Factors associated with metastatic involvement of level V.

Variable	Category	Level V Involvement, *n*/*N* (%)	*p*-Value
Sex	Female	8/40 (20%)	0.365
	Male	8/27 (29.6%)	
Age	≤45 years	10/34 (29.4%)	0.281
	>45 years	6/33 (18.2%)	
Surgical setting	Initial surgery	11/43 (25.6%)	0.662
	Reoperation	5/24 (20.8%)	
Bethesda category †	II	2/6 (33.3%)	0.203
	V	6/15 (40%)	
	VI	8/42 (19%)	
EU-TIRADS †	4	5/16 (31.2%)	0.309
	5	7/38 (18.4%)	
Histological subtype *†	Classic PTC	15/45 (33.3%)	**0.027**
	Follicular variant	0/11 (0%)	
	Aggressive variants	1/10 (10%)	
BRAF status §	Mutated	2/16 (12.5%)	0.602
	Wild-type	2/9 (22.2%)	
Multicentricity †	No	10/36 (27.8%)	0.463
	Yes	6/30 (20%)	
Extrathyroidal extension †	No	12/46 (26.1%)	0.758
	Yes	4/20 (20%)	
Lymphocytic thyroiditis	No	13/53 (24.5%)	1.000
	Yes	3/14 (21.4%)	
Global LNR	≤0.25	6/34 (17.6%)	0.224
	>0.25	10/33 (30.3%)	

* Histological subtype was available for 66 of the 67 patients with evaluable level V status. † Analyses were performed using available data for each variable; therefore, denominators may differ. § BRAF status was analyzed based on available cases only (*n* = 25). Note: Values are *n*/*N* (%) unless otherwise indicated. *p*-values were obtained using Pearson’s χ^2^ test, Fisher’s exact test or Fisher-Freeman-Halton exact test, as appropriate. LNR, lymph node ratio. Bold values indicate statistical significance (*p* < 0.05).

## Data Availability

The data supporting the findings of this study are not publicly available due to patient privacy and data protection restrictions. Access to the data may be considered upon reasonable request, subject to the applicable ethical, institutional, and Eurocrine^®^ Registry data governance requirements.

## Abbreviations

AHNS	American Head and Neck Society
ATA	American Thyroid Association
CI	Confidence Interval
ENE	Extranodal Extension
ETE	Extrathyroidal Extension
EU-TIRADS	European Thyroid Imaging Reporting and Data System
IQR	Interquartile Range
LNR	Lymph Node Ratio
OR	Odds Ratio
pN1b	Pathological N1b nodal classification
PTC	Papillary Thyroid Carcinoma
TKI	Tyrosine Kinase Inhibitor
TNM	Tumor, Node, Metastasis
